# Multistep Approach Points to Compounds Responsible for the Biological Activity and Safety of Hydrolates from Nine *Lamiaceae* Medicinal Plants on Human Skin Fibroblasts

**DOI:** 10.3390/antiox12111988

**Published:** 2023-11-09

**Authors:** Katarina Smiljanić, Ivana Prodić, Sara Trifunovic, Maja Krstić Ristivojević, Milica Aćimović, Jovana Stanković Jeremić, Biljana Lončar, Vele Tešević

**Affiliations:** 1 University of Belgrade—Faculty of Chemistry (UBFC), Studentski Trg 12–16, 11158 Belgrade, Serbia; krstic_maja@chem.bg.ac.rs (M.K.R.); vtesevic@chem.bg.ac.rs (V.T.); 2 Institute of Virology, Vaccines and Sera “Torlak”—National Institute of the Republic of Serbia, Vojvode Stepe 458, 11152 Belgrade, Serbia; iprodic@torlak.rs; 3 Institute of Molecular Genetics and Genetic Engineering (IMGGE), University of Belgrade, Vojvode Stepe 444a, 11042 Belgrade, Serbia; strifunovic@imgge.bg.ac.rs; 4 Mediterranean Institute for Life Sciences, 21000 Split, Croatia; 5 Institute of Field and Vegetable Crops—National Institute of the Republic of Serbia, 21101 Novi Sad, Serbia; milica.acimovic@ifvcns.ns.ac.rs; 6 Institute of Chemistry, Technology and Metallurgy, National Institute of the Republic of Serbia, University of Belgrade, 11000 Belgrade, Serbia; jovanas@chem.bg.ac.rs; 7 Faculty of Technology Novi Sad, University of Novi Sad, 21000 Novi Sad, Serbia; cbiljana@uns.ac.rs

**Keywords:** anti-proliferative effects, hydrolates, natural cosmetics, proliferative effects, skin fibroblast viability, STITCH database, volatile organic compounds

## Abstract

As byproducts of essential oil distillation, hydrolates are used in natural cosmetics/biomedicine due to their beneficial skin effects. However, data on their safety with relevant biological targets, such as human skin cells, are scarce. Therefore, we have tested nine hydrolates from the Lamiaceae family with skin fibroblasts that are responsible for extracellular collagenous matrix builds. Thyme, oregano, and winter savoury hydrolates showed several times higher total phenolics, which correlated strongly with their radical scavenging and antioxidative capacity; there was no correlation between their viability profiles and the reducing sugar levels. No proteins/peptides were detected. All hydrolates appeared safe for prolonged skin exposure except for 10-fold diluted lavender, which showed cytotoxicity (~20%), as well as rosemary and lavandin (~10%) using viability, DNA synthesis, and cell count testing. Clary sage, oregano, lemon balm, and thyme hydrolates (10-fold diluted) increased fibroblast viability and/or proliferation by 10–30% compared with the control, while their viability remained unaffected by Mentha and winter savoury. In line with the STITCH database, increased viability could be attributed to thymol presence in oregano and thyme hydrolates in lemon balm, which is most likely attributable to neral and geranial. The proliferative effect of clary sage could be supported by alpha-terpineol, not linalool. The major volatile organic compounds (VOCs) associated with cytotoxic effects on fibroblasts were borneol, 1,8-cineole, and terpinene-4-ol. Further research with pure compounds is warranted to confirm the roles of VOCs in the observed effects that are relevant to cosmetic and wound healing aspects.

## 1. Introduction

Since ancient times, cosmetic products have been used for their health benefits and to enhance beauty or body odour [1]. There is growing interest in cosmetic products that are safe for human skin [2], especially ones of natural origin. Moreover, natural cosmetic products are a new global trend wherein consumer preferences focus on ecology and sustainability [3]. Considering that natural cosmetics are a fast-growing segment of the cosmetics industry, there is a great demand for natural raw materials [4] but also an in-depth consideration of their safety profiles on relevant biological targets.

Essential oils are very popular in cosmetics, perfumes, and household products because of their pleasant odour and wide range of biological activities [5,6]. For example, peppermint (*Mentha piperita*) (MP) essential oil contains menthol and menthone as main components, which possess a fresh, sharp menthol odour and a pungent taste followed by a cooling sensation. Peppermint is widely used in perfumes, lipsticks, face and body creams, shampoos (as a scalp-healing treatment and for hair growth conditioning), shaving creams and foams, after-shave lotions, etc. [6,7,8,9,10]. Rosemary (*Rosmarinus officinalis*) (RO) essential oil mainly contains 1,8-cineole, camphor, and borneol in high percentages, which are the sources of the balsamic, woody, camphoraceous, and slightly minty odour. It is used for massages (to produce hyperaemia) and in cosmetic formulations for treating cellulite, alopecia, ultraviolet damage, and ageing [6,11,12]. Lavender (*Lavandula angustifolia*, formerly known as *L. officinalis*) (LO) and lavandin (*L. intermedia*) (LI) contain two main constituents in their essential oils, linalool and linalyl acetate, which give them characteristic floral, herbal, and clove-like odours [13,14,15]. Lavender is widely used in perfumes, cosmetics (in anti-acne skin products), and cleaning products, such as soaps, shampoos (for hair growth conditioning), detergents, etc. [4,6]. Clary sage (*Salvia sclarea*) (SS) essential oil has a similar composition to LI and LO as well as application [16]. Lemon balm (*Melissa officinalis*) (MO) essential oil has commercial value due to its characteristic lemony scent, originating from the main volatile compounds geranial, neral, citronellal, and geraniol [17]. Its essential oil is included in products that protect the skin against light and screen exposure damage and oxidative stress [18,19]. Thyme (*Thymus vulgaris*) (TV), winter savoury (*Satureja montana* ssp. *variegata*) (SM), and oregano (*Origanum vulgare*) (OV) essential oils mainly contain thymol and carvacrol as well as p-cymene and γ-terpinene [20,21,22], which are the sources of the characteristic pungent, warm odour used in cosmetics as a deodorant active agent. Their application is noted in biomedicine, hygiene, and skin care products [6,23].

As byproducts of essential oil distillation, hydrolates are important in natural medicine because of their antimicrobial, antioxidant, anti-cancer, and anti-inflammatory activities [24,25,26]. Literature reviews have shown that hydrolates obtained from nine selected plants from the *Lamiaceae* family have similar chemical compositions to their corresponding essential oils [27,28,29,30,31,32]. This presents the opportunity for applying hydrolates for the same purposes as essential oils. However, hydrolates, primarily aqueous solutions, can contain glycosides, sugar, small proteins, and peptides in traces that are not pertinent to the content of the essential oils. Hydrolates are used as replacements for the water phase in cosmetic products such as face tonics (which can be sprayed directly on the skin for a refreshing and hydrating effect), face masks, and body and hair care products [24,33].

Despite numerous studies dedicated to antioxidative, antibacterial, and anti-cancer properties (more than a hundred) [26,34], the literature addressing the question of hydrolates safety and biological activity on relevant cellular constituents of the skin (primary cultures of fibroblasts and/or keratinocytes) is scarce. Only two studies address the safety of hydrolates, chamomile vacuum-derived hydrolate on primary dermal fibroblasts [35] and *Vitis vinifera* hydrolate on commercial gingival fibroblasts to assess cytotoxicity [36]. Until now, sugars, glycosides, and proteinaceous content have not been evaluated in any hydrolates except for the glycosides in the total, spent, and residual water of three *Lamiaceae* species (thyme, oregano, and basil) [37]. There is a possibility that small proteins (≤10 kDa), their fragments, or small peptides (~1500 Da) are present and affect their biological properties, which are mistakenly attributed to well-studied volatile organic compounds (VOCs) in hydrolates.

Therefore, this study aimed to evaluate the biological safety and activity of hydrolates in the primary cell cultures of human skin fibroblasts, and to characterise chemical and antioxidative properties of hydrolates obtained from nine *Lamiaceae* medicinal plants, mainly used for essential oil extraction and suitable for growing in continental climate.

## 2. Materials and Methods

### 2.1. Plant Materials

Nine medicinal plants from the *Lamiaceae* family, *M. piperita* (MP), *R. officinalis* (RO), *L. officinalis* (LO), *T. vulgaris* (TV), *S. sclarea* (SS), *S. montana* ssp. *variegata* (SM), *L. intermedia* (LI), *O. vulgare* (OV), and *M. officinalis* (MO), were cultivated at the Department of Vegetable and Alternative Crops of Bački Petrovac, Institute of Field and Vegetable Crops, Novi Sad, Serbia. Hydrolates were obtained as byproducts during the steam extraction of essential oils of aerial plant parts in a pilot-scale distillation unit with a constant volume of 0.8 m^3^. After the processes were complete, hydrolates were collected in Florentine flasks, filtered through filter paper, poured into sterile plastic bottles, and kept in a cool and dark place until further analysis. No external additives or preservatives were added that could impact the original pH values [38].

### 2.2. Chemicals

Organic solvents were purchased from J.T. Baker (Mallinckrodt Baker, Phillipsburg, NJ, USA). Foetal bovine albumin, L-glutamine, penicillin, 3-(4,5-dimethylthiazol-2-yl)-2,5-diphenyltetrazolium bromide (MTT), and all other chemicals, unless otherwise stated, were purchased from Sigma (Sigma, Darmstadt, Germany). HPLC-grade water (conductivity of 0.055 μS/cm and resistance of 18.2 MΩ/cm at 25 °C) was prepared using a Smart2Pure3™ purification system (ThermoFisher Scientific, Waltham, MA, USA).

### 2.3. Gas Chromatography Coupled with Mass Spectrometry Analyses of Volatile Compounds in Hydrolates

Volatile compounds were extracted from 400 mL of hydrolate using CH_2_Cl_2_ by a Likens–Nickerson apparatus for 2 h. Volatile components were determined by gas chromatography coupled to flame ionisation and mass spectrometry detectors (GC/FID and GC/MS) using an Agilent 7890A GC system, which was equipped with a non-polar capillary MS column HP-5 (30 m × 0.25 mm, 0.25 μm). The components were identified according to their linear retention indices and through a comparison with MS libraries. The relative abundance of each detected component was calculated from the GC/FID chromatograms as a percentage by dividing the area of each peak by the sum of the areas of all peaks (only identified components are shown).

### 2.4. Total Phenolic Content (TPC) Analyses and Biological Activity Tests

#### 2.4.1. TPC

The method used to determine the concentration of water-soluble phenolics in plant extracts was a modified Folin–Ciocalteau protocol [39] adjusted for measurements in 96-well microtiter plates [40].

Hydrolates were filtered through 0.22 μm PES filters, and 100 μL of 10 time-diluted Folin–Ciocalteau reagent was added to 10 μL of hydrolates. The reaction was left in the dark at room temperature to incubate for 5 min. After that, 140 μL of freshly produced 7.5% Na_2_CO_3_ was added, and the mixture was incubated in the dark for 90 min. The absorbance of the mixture was measured at 620 nm. All measurements were conducted in triplicates. Total phenolics were expressed as milligrams of gallic acid equivalents (GAE) per L of hydrolate using a standard calibration curve constructed of different concentrations of gallic acid with a linearity ranging between 0.01 and 0.5 mg/mL of gallic acid.

#### 2.4.2. Antioxidative Test with 2,2-Diphenyl-1-picrylhydrazyl Compound (DPPH Assay)

The antioxidant activity of undiluted hydrolates previously filtered through 0.22 μm PES filters was assessed by a DPPH assay. The decrease in the DPPH absorbance after 24 h in the dark was measured and compared with a blank. In brief, 10 μL of the antioxidant solution was mixed with 190 μL of 6 × 10^−5^ mol/L of DPPH and incubated for 24 h in the dark at room temperature. Afterwards, the absorbance at 540 nm was measured, and the scavenging ability was calculated against methanol as a blank. This analysis was carried out in a triplicate, and the results are expressed as the means of % inhibition of the DPPH radical, which was calculated as follows: % inhibition = ((Abs control − Abs sample)/Abs control) × 100 [41]. In addition, the radical scavenging ability was expressed through the standard reference data that was obtained using the water-soluble synthetic analogue of vitamin E, Trolox (6-hydroxy-2,5,7,8-tetramethylchroman-2-carboxylic acid), with the standard curve of 12.5–400 µM serving as the concentration equivalent of the Trolox standard used in the assay. The Trolox equivalent of antioxidant capacity (TEAC) is the millimolar concentration of Trolox having an antioxidant capacity equivalent to 1 mM of the tested compound [42].

#### 2.4.3. Antioxidative Test with 2,2-Azino-bis(3-ethylbenzothiazoline-6-Sulfonate) Cation (ABTS+ Assay)

The protocol applied the method of Re et al. [43] with some modifications. ABTS+ solution (7.0 mM) and 245 mM of ammonium persulfate (APS) solution were equally mixed so that the final APS concentration was 2.45 mM and was allowed to react for 12 h at room temperature in the dark. Afterwards, the solution was diluted to an appropriate volume of ultrapure water to obtain an absorbance of 0.7 units at 734 nm. Fresh ABTS+ solution was prepared for each assay. Undiluted hydrolates (10 μL, previously filtered through 0.22 μm PES filters) were allowed to react with 190 μL of the ABTS+ solution for 5 min in the dark, after which the absorbance was measured at 734 nm. The percentage inhibition of the absorbance at 734 nm was calculated as the means of % inhibition ± the SD of the ABTS+ radical as per the following calculation: % inhibition = ((Abs control − Abs sample)/Abs control) × 100. In addition, antioxidative power was expressed through the standard reference data obtained using the Trolox standard curve of 12.5–400 µM, which served as the concentration equivalent of the Trolox standard used in the assay.

### 2.5. Colorimetric Test for Determination of Reducing Sugars and Polyols Content

The test was conducted based on the Dubois protocol for determining reducing sugar levels [44], as modified for 96-well plates. Briefly, 40 µL of hydrolate, previously filtered through 0.22 μm PES filters, was dispensed into the microtiter plate, followed by the addition of 20 µL of 5% phenol solution in water (freshly prepared) and 100 µL of concentrated H_2_SO_4_. Readings were performed after 10 min of incubation at room temperature at 492 nm. The standard curve was generated with various glucose concentrations (0.1–1.0 mg/mL), and all samples were performed in triplicates.

### 2.6. MTT, BrdU Viability, and Cell Count Tests of Primary Human Skin Fibroblast Culture

Primary human dermal fibroblasts were a kind gift from the Mediterranean Institute for Life Sciences, which were obtained from a healthy, 24-year-old man. Cells were cultured in Dulbecco’s Modified Eagle Medium (Sigma) supplemented with 10% (*v*/*v*) Foetal Bovine Serum (Gibco, Waltham, MA, USA) at 37 °C in a humidified 5% CO_2_ atmosphere. Their total life span was 56 ± 2 days or 27 ± 1 passages.

MTT assay tests were performed on cells from passage 14 to passage 16. Cells were seeded in 96-well plates at 4 × 10^3^ cells per well and treated 24 h after seeding. After 48 h of treatments, which included incubation with a 10%, 5%, and 2.5% extent of each hydrolate in triplicates in liquid cell medium, thiazolyl blue tetrazolium bromide (MTT) (Sigma) was added and incubated at 37 °C and 5% CO_2_ for 2 h. MTT formazan crystals were dissolved in dimethyl sulfoxide (DMSO), and absorbance was measured at 570 nm. Samples were normalised to the untreated control.

The DNA synthesis was assessed as previously explained [45] by measuring the incorporation of 5-bromo-29-deoxyuridine (BrdU) into primary dermal fibroblasts using the BrdU proliferation kit (Roche Diagnostics, Penzberg, Germany). Quiescent cells cultured in 96-well plates were treated with hydrolates for 46 h and then incubated with BrdU (10 mM) for 2 h. Fibroblasts were fixed, and BrdU was labelled with a peroxidase-conjugated anti-BrdU antibody, which was followed by the addition of a peroxidase substrate. The reaction was terminated by adding 30 μL of 1 M H_2_SO_4_, and the optical density was measured at 450 nm. The cell number in experimental fibroblast groups was checked using the crystal violet-staining technique in 96-well plates by measuring the optical density at 570 nm. For each experiment, standard curves were produced with the known cell numbers. These data were confirmed with the cell count in the Neubauer hemocytometer using the trypan blue staining method.

### 2.7. Protein and Peptides Analyses in Hydrolates

To assess the presence of proteinaceous content, hydrolates were filtered through hydrophilic, polyether sulfone-PES syringe filters with a 0.22 μm pore size (Membrane-Solutions, Auburn, WA, USA). For the assessment of proteins and peptides presence, 1 mL of filtered hydrolates was concentrated until the liquid had evaporated entirely in a vacuum by SpeedVac at room temperature (Eppendorf, Germany). On the other hand, 10 mL of hydrolates were mixed with four volumes of 13% trichloroacetic acid (TCA) in acetone for the precipitation of potentially present proteins >5 kDa [46]. Protein and peptide content was assessed using a bicinchoninic acid (BCA) assay and a reducing, one-dimensional, 16% sodium dodecyl sulphate polyacrylamide gel electrophoresis (1D SDS-PAGE) method according to the Laemmli protocol [47]. Pierce™ Unstained Protein molecular weight marker mixtures of 14.4, 18.4, 25, 35, 45, 66.4, and 116 kDa proteins were applied as a means of positive and quantitative protein control (7 μL of mixture secures 1 μg of each protein, visible as a distinct band).

### 2.8. Statistical Analysis

One-way and ordinary two-way ANOVA tests, including the post tests of multiple comparisons such as Dunnett’s and Sidak’s tests, were undertaken using GraphPad Prism 7.0 software (GraphPad, San Diego, CA, USA) to analyse the data of three types of fibroblast viability testing. Also, GraphPad Prism software was used for the Pearson correlation of antioxidant testing and TPC. Principal component analysis (PCA) was used as a pattern recognition technique with the PLSToolbox, v.6.2.1, for MATLAB 7.12.0 (R2011a). The PCA was performed using a singular value decomposition algorithm and a 0.95 confidence level for the Q and T2 hoteling limits for outliers.

## 3. Results and Discussion

### 3.1. Chemical Composition of VOCs in Hydrolates

Hydrolates (hydrosols) are aqueous-based solutions or condensates that are separated from the essential oil during their production by steam distillation, not to be confused with the “residual water” that remains in the distillation vessel. Hydrolates usually contain less than 0.1% volatile organic compounds (VOCs) [26], with a range of 0.3–1.2 g/L [48,49]. All nine hydrolates fall above the VOCs extent range (Table S1).

We emphasise the importance of a comprehensive characterisation of the hydrolate content if one wants to understand which components are responsible for specific biological effects in hydrolates. For example, a hypothetical hydrolate has up to 1 g of VOCs per litre, corresponding to a concentration of 0.1%, and if a particular molecule accounts for only 0.1% of the total VOCs, its resulting concentration is 0.0001 g/L. When assuming the averaged molecular mass of the compounds from Table 1 to be 150 u, we obtain a molar concentration of ~0.7 μM, which is a relevant concentration concerning receptor–agonist interactions [50]. A list of identified chemical components in the hydrolates used in this study is shown in Table 1. Some dominant compounds of the nine selected hydrolates were already reported, such as *T. vulgaris* hydrolate (TV) with thymol (73.6%), which is the same as the extent in the study of Konstantinović et al. [51]; in SS hydrolate, linalool (63.3%) and α-terpineol (26.8%) were reported in the study of Aćimović et al. [16]. In addition, the main component in SM hydrolate was carvacrol (96.4%) [52], while in LI hydrolate, it was linalool (26.0%), borneol (24.4%), 1,8-cineole (12.7%), and terpinen-4-ol (12.2%) [15].

The other five hydrolates from plants grown in Serbia have not yet been comprehensively characterised with respect to their water-soluble volatile organic compounds until now. In the LO hydrolate, 39 different compounds were detected (Table 1), with borneol (21.8%) and menthol (20.1%) being the predominant components, in contrast to *Lavandula angustifolia* hydrolate grown in Tuscany (Italy), which predominantly had linalool (43%), camphor (18%), and *α*-terpineol (12%) out of only 7 molecules identified in the VOC fraction [53]. All of these components were minor VOC constituents in the LO hydrolate of Serbian origin. Such drastic differences in VOC profiles can be partially explained by differences in the experimental approach, with the current study using an enrichment step VOC by a liquid–liquid extraction. It is important to note that the LO hydrolate VOC identification rate was below 90%, e.g., 85% (Table 1). In contrast to the LO hydrolates comparison, the difference between RO hydrolates was less pronounced, with camphor (29.2%), menthol (17.8%), 1,8-cineole (12.6%), and borneol (10.1%) dictating the VOC nature of the hydrolate from Serbia (31 volatile molecules were quantified, see Table 1). In comparison, 1,8-cineole (56%), camphor (20.3%), and borneol (10.1%) were dominant in the Italian RO hydrolate [53].

In the MO hydrolate of the current study, carvacrol (28%), neral (22.6%), and geranial (20.5%) were the dominant VOCs among the 39 components quantified (Table 1), which is very similar to the findings of Collin et al. [48] and is in contrast to lemon balm hydrolate from Persia, which is used to ameliorate feminine hormonal remedies [54]. Namely, the significant molecules dictating the VOC profile were thymol (47%) and carvacrol (30%), while each of the remaining 11 identified compounds was in low abundance (<5% of the VOC share) [54].

In MP hydrolate, menthol (30.6%), menthone (14.4%), and 1,8-cineole (12.8%) were dominant (Table 1), similar to the published results [30,55]; however menthol was twice less abundant and there was a more versatile VOC portfolio than in the mentioned studies. In OV hydrolate, the main components of VOCs were carvacrol (65.5%) and thymol (12.3%) among the 27 identified VOCs (Table 1), in contrast to Popa et al. [56], where 1-octen-3-ol (13%), caryophyllene oxide (12%), and linalool (12%) predominated; this difference could be caused at least in part by the air-drying before the distillation process.

### 3.2. Antioxidative Properties, Total Phenolics, and Reducing Sugar Levels of Hydrolates

Table 2 presents the averaged pH measurements of hydrolates batches that do not contain preservatives. In their intact state, most of them were acidic—especially both of the lavender hydrolates (LO and LI)—while muscat sage (SS), MO, and winter savoury (SM) were neutral. This finding is in line with the literature data [34]. In addition, pH values of pure DMEM cell culture medium and those with a 10% inclusion of hydrolates before and after 48 h treatment were measured to exclude the possibility that a substantial decrease or change in environmental pH could affect fibroblast viability and proliferation [57]. Even though LI hydrolate was the most acidic within the group (Table 2), its 10% inclusion did not affect the neutral pH of 7.40 of the cell culture medium.

Determining the concentration of reducing sugars, especially glucose, is essential if hydrolates are used in pure form in cosmetics or are added substantially to meals and beverages from a functional food point of view. This is because a high concentration of chronic glucose exposure, as in diabetes mellitus (above 1240 mg/L or 6.9 mM), impairs primary skin fibroblasts in wound healing and delays cell migration [58] and proliferation [59]. However, in this specific experimental setup with primary cell cultures of dermal fibroblasts and acute exposure, the obtained sugar concentrations of the hydrolates (Table 2) could not be effective as their level is far below the level of glucose used in the liquid cell culture medium (4500 mg/L, 25 mM), which is close to the 5400 mg/L glucose (30 mM) employed by Xuan et al. [58] to simulate high glucose level effects on wound healing. Nevertheless, if they are to be applied without dilutions and directly on the skin with the instruction to be left on it, caution should be taken when the values exceed the upper limit of the normal capillary glucose range (above 1100 mg/L), such as in the case of LI hydrolate (1247 ± 99 mg/L, Table 2). Therefore, further proteomic studies that comprehensively examine the differences in fibroblast expression profiles with prolonged (chronic), higher glucose treatment (6.5–12 mM) would be desirable, especially those employing an approach similar to the study of Trifunovic et al. [60], which examined the effects of electronic cigarette liquids.

TV, SM, and OV hydrolates dominate the rest regarding antioxidant activity, with DPPH radical scavenging activities of 49%, 37%, and 36% compared with methanol blank; moreover, all three were above 500 μM as expressed in the TEAC for inter-study comparisons (Table 2). A similar result was replicated when assessing the antioxidative capacity with an ABTS^•+^ assay but with no differences observed between the three (~470 μM of TEAC, around 90% of decolourisation, see Table 2). The explanation for the several times higher radical scavenging activity and antioxidant capacity of TV, SM, and OV over the rest of the hydrolates is most probably in their several times higher content of total phenolics compared with the rest of the hydrolates (Table 2). In support of this is a solid, positive correlation between these variables in the Pearson correlation analyses; the ABTS assay is positively correlated to the DPPH assay (*r* = 0.927 at *p* ≤ 0.001), the TPC content is positively correlated to the DPPH assay (*r* = 0.96; *p* ≤ 0.0001) and is positively correlated to the ABTS assay (*r* = 0.952; *p* ≤ 0.001) (Table S2).

When the results of the antioxidant activity of hydrolates are expressed as mM or μM of the TEAC, it is easy to observe and comprehend their potential within the antioxidant context. For example, both test values of TV, SM, and OV can be considered as substantial free radical scavenging activity and antioxidative capacity when compared with the antioxidant activity of 82 pure substances, among which 1 mM of vitamins C and B9 have shown the highest TEAC values as performed with the DPPH assay (267 μM and 230 μM, respectively) [61]. In this sense, it would be a reasonable idea to express the antioxidant activity (strength) of hydrolates as milli- or micromoles of Trolox equivalents per L of hydrolate instead of the various [62,63] and somewhat confusing [64] formats found in the literature.

Regarding the comparison of data from the literature on the antioxidant activity researched by DPPH and ABTS tests of the same plant species and their hydrolates obtained by steam distillation as in our study, SM (Serbia), LO (Bulgaria, France), MO (Bulgaria), MP (Bulgaria), RO (France), SS (Slovak Republic), and LI and OV (Romania) hydrolates [38,56,63,65] are in the range of agreement with our results (Table 2). However, TV could not be appropriately compared with other TV hydrolates due to the different formats used in the studies [62,66,67] except for the excellent data agreement present for TV hydrolates from Romania [56].

### 3.3. Hydrolates and Proteinaceous Content Characterisation

In the distillation process, the volatile organic compounds first evaporate and then condense with water to form the two-layer distillate; the hydrolates are almost pure water. Vitamins, minerals, amino acids, tannins, flavonoids, carotenoids, alkaloids, and many other compounds generally do not evaporate. However, the intense evaporation of water during distillation can result in larger molecules and particles being trapped in the aerosol of the water vapour droplets, where they travel to the condenser and eventually end up in the distillate [68]. The amount of distilled non-volatiles is probably small or present in traces, but this needs to be confirmed experimentally before concluding that they are irrelevant to the biological activity of hydrolates.

Besides the VOCs and polyphenolics extensively characterized in hydrolates, we could not find any publication that aimed to reveal the presence of proteinaceous content in them, even though some proteins and peptides function as mitogens, activating epidermal growth factor receptor (EGFR), the most critical pathway for triggering proliferation in skin cells, including fibroblasts [45,69]. There were no examples of plant-based EGFR agonists until the recently discovered 36-residue long peptide “bleogen pB1” from the Cactaceae family [70], which mimics the effects of the endogenous agonist epidermal growth factor (EGF). In line with this, we searched for peptides and proteins in the nine Lamiaceae hydrolates.

**Figure 1:**
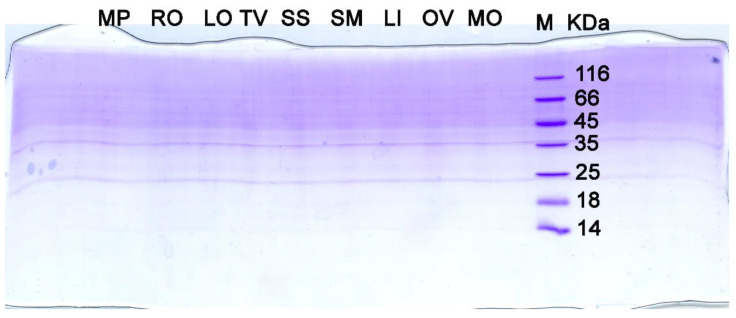
Electrophoretically resolved sodium dodecyl sulphate polyacrylamide gel (16%) with 1 mL of hydrolates concentrated on SpeedVac (room temperature under vacuum) until a 10 μL volume, mixed with 10 μL of 2 × Laemmli buffer in denaturing conditions and stained with CBB 250-R. *M. piperita* (MP), *R. officinalis* (RO), *L. officinalis* (LO), *T. vulgaris* (TV), *S. sclarea* (SS), *S. montana* ssp. *variegata* (SM), *L. intermedia* (LI), *O. vulgare* (OV), and *M. officinalis* (MO). M—protein weight markers in kilodaltons (kDa). Each marker band in the M lane represents 1 μg of protein.

Proteins/peptides were not detected at a ≥ 0.1 ppm level (Figure 1), including proteins larger than 5 kDa at a ≥ 0.01 ppm level (Table S3, Figure S1). The former and latter cases encompass a nanomolar concentration range of EGF (1.5–15 nM), eliciting the usual cellular responses in addition to the lower picomolar concentration range of EGF, which is known to bind its receptor on high-affinity sites, leading to mitogenic effects [71]. It is desirable to study in more depth for the presence of small, bioactive peptides as 0.1 ppm and 0.01 ppm concentrations are not “diluted” enough to confidently exclude the existence of small bioactive peptides (≤5 kDa). Thus, with great caution, we can say that proteins and peptides do not contribute to the observed hydrolate characteristics.

### 3.4. Hydrolates Exert Differential Biological Activity and Safety Outcomes on the Primary Culture of Human Skin Fibroblasts

**Figure 2:**
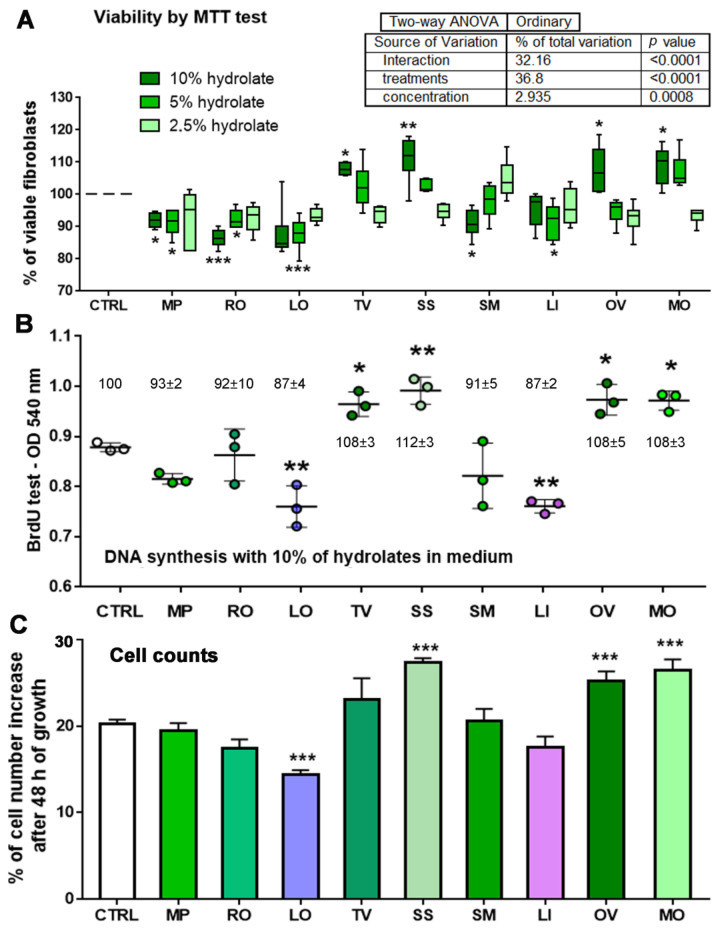
Viability assessments and cell counts of primary human skin fibroblasts treated with nine plant hydrolates. (**A**) MTT assay with three different hydrolates concentrations in a primary cell culture medium. (**B**) DNA synthesis assessment by BrdU testing with the highest concentration point (10%) applied in the MTT test. Results are expressed as optical density values and above or under them as a percentage in relation to the control (average ± SD). (**C**) The number of cells before and after the treatment was obtained by crystal violet and trypan blue staining and counting. A two-way ordinary ANOVA test was applied in (**A**) and one-way ANOVA with Dunnett’s multiple comparison tests were applied in (**B**,**C**) to show significant differences among different hydrolates treatments compared with the control. All tests were conducted with three biological batches, each in triplicates. *** denotes a difference at *p* < 0.0005; ** denotes a difference at *p* < 0.005; * denotes a difference at *p* < 0.05. *M. piperita* (MP), *R. officinalis* (RO), *L. officinalis* (LO), *T. vulgaris* (TV), *S. sclarea* (SS), *S. montana* ssp. *variegata* (SM), *L. intermedia* (LI), *O. vulgare* (OV), and *M. officinalis* (MO).

MTT and BrdU tests, usually employed to study cytotoxicity, were used in this study with up to the maximum possible extent of hydrolates permitted in a cell culture medium (10%) as no significant cytotoxic effects were anticipated. This is similar to the wound healing study and human skin fibroblast proliferation enhancement with quince seed mucilage [72]. Four hydrolates, TV, SS, OV, and MO, have shown an increased viability of primary skin fibroblasts compared with the untreated control cells (Figure 2).

These beneficial effects of SS, OV, and MO, the opposite of the cytotoxicity trend, were illustrated by a significant increase in cell viability by the MTT test, DNA synthesis by the BrdU test, and a concomitant increase in cell counts, meaning that an increase in fibroblasts proliferation is likely possible. A particular case of beneficial viability enhancement is demonstrated with TV hydrolate, where a significant increase in cell counts is absent; however, MTT and BrdU tests show increased fibroblast viability (Figure 2), pointing to other potentiated viability aspects, such as increased growth or metabolic state (e.g., increased production of extracellular matrix components). As a positive control, cells were treated with 10 nM of recombinant human EGF (hrEGF) for 48 h, and a 1.5 times higher proliferation was obtained compared with the control cells by the BrdU test (Figure S2), similar to previous studies [73,74].

In contrast to the enhancement observed with hrEGF and four hydrolates, the 10% extent of LO hydrolate exerted a slight (~10%) but significant decrease in viability, including a reduction in cell number compared with the control and pointing to decreased proliferation. In addition, the highest concentrations of RO and LI showed, to a lesser extent, the suppressive viability effects on fibroblasts, though the parameters were not significant in all three tests (Figure 2). Therefore, it would be desirable to characterise the cell cycle and proliferation rate using the flow cytometry approach to confirm the observed effects and reveal the mechanisms by which specific hydrolates promote or suppress fibroblast proliferation in the fashion described by Krstić et al. [75]. LO hydrolate’s VOC identification rate was the lowest (85%) and, as noticed previously, was the only hydrolate showing a slight but significant decrease in metabolic activity/proliferation of primary human cell fibroblasts in all three viability tests employed. It remains to be discovered if this 15% of the unknown VOC could be responsible for the effects observed. The rest of the hydrolates did not exert any measurable viability/cytotoxic effects on skin fibroblasts; therefore, we can conclude that they are safe for use in skin treatments, including the lowest concentration of RO and LI. In addition, TV, SS, OV, and MO are beneficial and desirable due to the expected beneficial effects on the skin texture as skin fibroblasts are the leading producers of collagen and other components of the extracellular skin matrix. Caution should be taken when implementing *L. angustifolia*, formerly *L. officinalis*, into skin cosmetic preparations, where this hydrolate should not exceed 2.5% in the total skin formulation. All of these anticipated beneficial effects of TV, SS, OV, and MO should be assessed in clinical study trials. At the same time, it would be interesting to include these hydrolates in the design of further wound healing studies to see if and how their action promotes healing processes.

### 3.5. Principal Component Analysis

**Figure 3:**
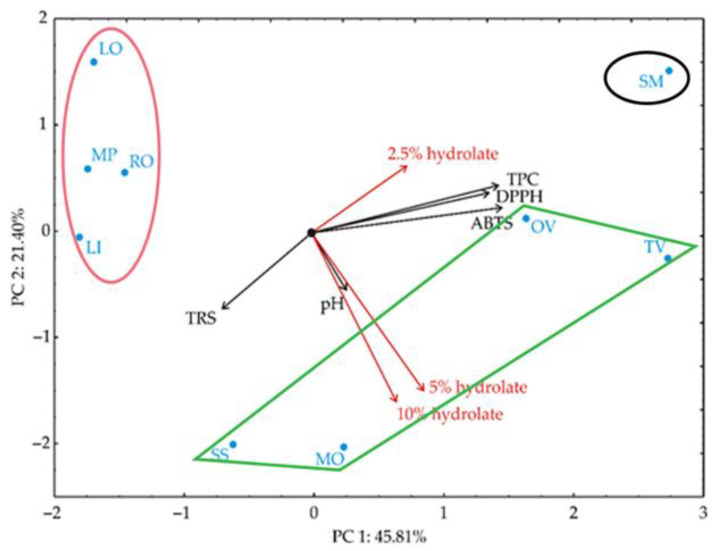
PCA ordination of variables based on component correlations. TPC—total phenolic content; TRS—total reducing sugars; DPPH—2,2-diphenyl-1-picrylhydrazyl assay; ABTS—2,2′-azino-bis(3-ethylbenzothiazoline-6-sulfonic Acid) assay. Red arrows denote viability testing by the MTT assay with different hydrolates concentrations. The red and black ellipses and the green distorted rectangles represent a separation of groups of hydrolates.

The hydrolates presented by points in Figure 3, which represent the graphical results of the PCA, are geometrically close to each other, indicating the similarity of patterns. The vector’s orientation, which describes the variable in factor space, indicates an increasing trend in these variables. The angles between corresponding variables indicate the degree of their correlations (small angles corresponding to high correlations) (Figure 3).

The PCA of the presented data explained that the first two components accounted for 67.21% of the total variance in the eight variables (antioxidant activity of hydrolates, their total phenolic and reducing sugar contents, and the primary human skin fibroblast viability test). Considering the map of the PCA performed on the data, the value of TPC (which contributed 24.9% of the total variance based on correlations), DPPH test (22.3%), ABTS (25.7%), and the 2.5% hydrolate content in the cell culture medium (9.0%) exhibited positive scores according to the first principal component (PC1) (Figure 3). On the other hand, the negative contribution to the second principal component calculation was observed for the total reducing sugars (TRS) content (8.0% of the total variance based on correlations), 5% hydrolate content (34.6%), and 10% hydrolate content (39.9%).

The PCA has revealed nothing new except that it confirms that there are three groups of hydrolates in terms of their biological effects on primary dermal fibroblasts. However, the question remains as to which component(s) in the hydrolates of clary sage (SS), lemon balm (MO), oregano (OV) and, in second place, thyme (TV), are responsible for the increased viability and proliferation of dermal fibroblasts. In addition, it remains to be elucidated which compound(s) in the lavender (LO), rosemary (RO), and lavandin (LI) hydrolates are responsible for the decreased fibroblast viability.

### 3.6. Relating the Structure of Major Hydrolates VOCs (≥10% Abundancy) with Biological Activity and Their Involvement in Protein Networking Using the STITCH Database

**Figure 4:**
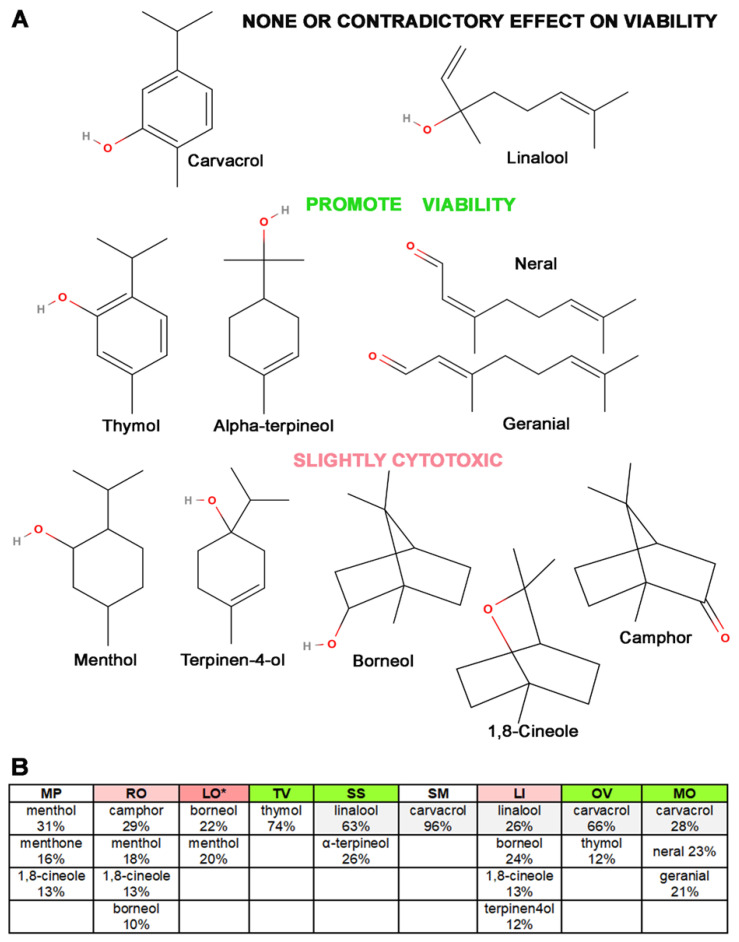
(**A**) Overview of the structural formulas of volatile organic compounds (VOCs) with abundance ≥ 10%, divided into three groups based on their exclusive presence in hydrolates that either promote or suppress fibroblast viability, including those with no or contradictory effects. (**B**) The abundance of up to the top five volatile compounds per hydrolate that each contribute at least a 10% share. Colours: grey shade—contradictory or no effects on the proliferation; * dark rose—significantly cytotoxic; pale rose—significantly cytotoxic for at least one of the three viability tests employed. MolView, a web application and intuitive, open-source program, was used to draw the chemical structural formulas (https://molview.org accessed on the 3 April 2023).

We evaluated the relationship between the structure and the potential biological effects of the major VOCs based on the viability profiles of their respective hydrolates on primary skin fibroblasts (Figure 4) by researching the known biological activity of the main VOCs contained in the hydrolates via the STITCH database http://stitch.embl.de/. The major VOC constituents were divided into three groups based on the viability effects exerted by their respective hydrolates:

(1) Major VOCs that likely do not contribute to the observed viability effects, such as linalool and carvacrol (the first row in Figure 4A);
(2) Major VOCs present solely in the hydrolates that exerted beneficial viability effects, such as α-terpineol, thymol, neral, and geranial (the second row in Figure 4A);
(3) Major VOCs present solely in the hydrolates that exerted observed cytotoxic effects, such as 1,8-cineole, camphor, menthol, menthone, borneol, and terpinen-4-ol, as shown in Figure 4.

**Figure 5:**
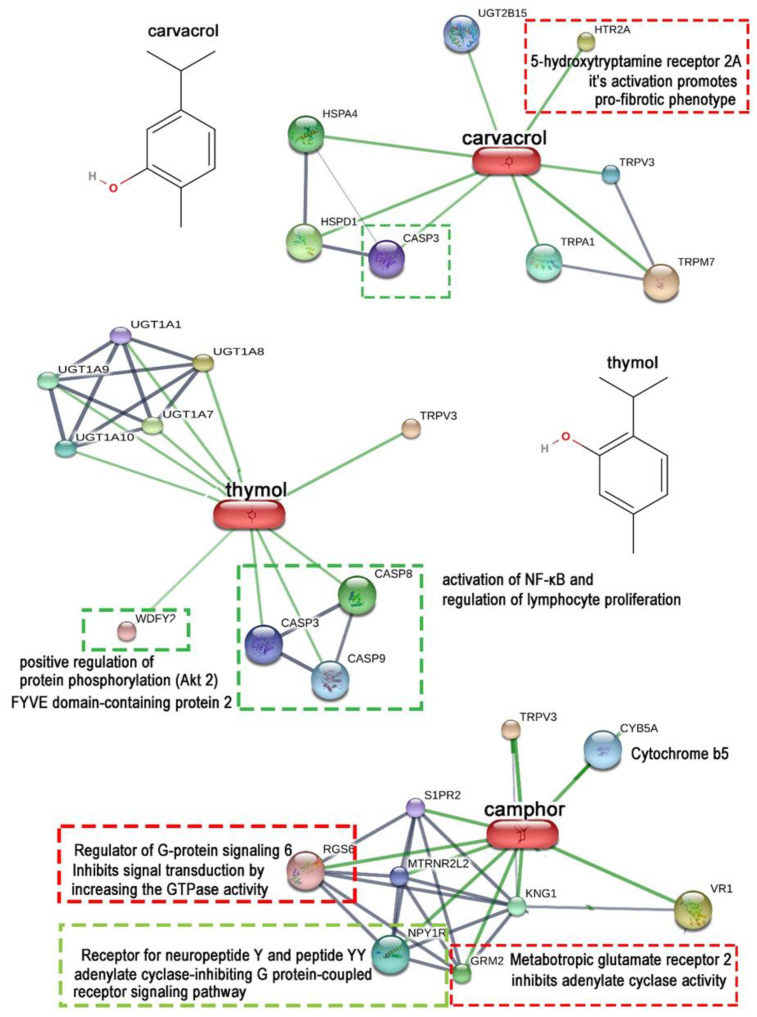
The confidence view of the protein networking of carvacrol (representative of the major VOCs from hydrolates that exert no net effect), thymol (representative of the major VOCs from hydrolates that exert a positive viability effect) and camphor (representative of the major VOCs from hydrolates that exert a negative viability effect), performed using the STITCH database http://stitch.embl.de/ accessed on the 9 April 2023. Only first-line protein targets of chemical compounds are shown with the host species of *Homo sapiens*. Thicker lines represent more robust associations. Green lines show chemical–protein interactions; protein–protein interactions are in grey, small and large node sizes differentiate between proteins with and without 3D structures, and coloured and white circles denote first- and second-line protein targets, respectively. The codes represent gene name abbreviations that code the designated proteins.

The apparent structural differences can be seen between the groups of the main VOCs found only in hydrolates that promote fibroblast viability and those found in hydrolates that exhibit mild cytotoxicity (Figure 4). While the former group possesses conjugated C-C double bonds (except alpha terpineol), the latter is almost devoid of double bonds and mainly comprises polycyclic compounds. Therefore, we must note that the subtitles in Figure 4A are related to the effects exerted by the hydrolates listed in Figure 4B and are not directly related to the major volatile organic compounds; they could be just one of the candidates responsible for the safety effects observed in primary skin fibroblasts. Regarding the first group of VOCs, termed as “none or contradictory effects”, carvacrol is present in SM hydrolate as the sole significant VOC; SM hydrolate does not exert any net effects on fibroblast viability. At the same time, carvacrol is present in OV and MO hydrolates, which contain thymol, neral, and geranial as major VOCs (Figure 4B), and these hydrolates promote fibroblast proliferation significantly. Linalool is another major VOC in two hydrolates (LI and SS) with opposite biological activity. Therefore, besides carvacrol, linalool is another major VOC that is excluded as a possible cause of the observed effects of the investigated nine Lamiaceae hydrolates on the skin fibroblasts. Also, it is interesting to note that thymol, a major VOC found exclusively in TV and OV hydrolates that promote fibroblast viability, is a structural isomer of carvacrol (Figure 4A and Figure 5).

Figure 5 shows the first-line protein functional partners of carvacrol, thymol, and camphor as the representatives of major VOCs belonging to the three hydrolate groups mentioned at the beginning of this section and in Figure 4. The structural formulas of carvacrol and thymol were repeated so as to underline how slight difference in their structures (carvacrol contains a hydroxyl group at the orto position of the benzene ring, whereas thymol contains a hydroxyl group at the meta position of the benzene ring) caused significant differences in their first-line protein networking. After an analysis of the biological annotations, all protein targets, those that could directly influence signalling pathways leading to fibroblast proliferation or cause abnormal phenotypes involving the potentiation of apoptotic processes, pro-fibrotic phenotype expressions, etc., were highlighted with green and red dash boxes, implying positive and negative outcomes on dermal fibroblast viability. In line with the carvacrol position within “the group with no or contradictory effects”, two opposing signalling pathways were activated. Caspase 3 (CASP3) has been identified as a potential target whose activation could not only lead to the execution of apoptotic processes but also to the potentiation of the proliferation [76,77], which would depend on the involvement of CASP8 and CASP9, which are not found as first targets for carvacrol compound in contrast to thymol (Figure 5).

On the other hand, the predicted activation of the 5-hydroxytryptamine 2A receptor (5HTR2A) by carvacrol could lead to the formation of a pro-fibrotic fibroblast phenotype [78], which is a non-desirable feature, even for cosmetic aspects. Linalool is a true enigma as it has diverging primary protein targets in the STITCH database http://stitch.embl.de/cgi/network.pl?taskId=3YjkjWUQxRff (accessed on the 10 April 2023), such as nitric oxide synthase 1 (NOS1 as a positive switch) and tumour protein p53 suppressor (TP53) and a weak activation of transient receptor potential cation channel subfamily M member 8 (TRPM8) as negative proliferative switches. Linalool is present in SS hydrolate (63%) and promotes proliferation, and LI hydrolate (28%) shows slight cytotoxic effects (Figure 4B). Nevertheless, the cytotoxicity of lavender essential oil and its major components linalyl acetate (51%) and linalool (35%) to human skin fibroblasts has been shown with a linalool concentration range of, e.g., 0.005–0.08% (*v*/*v*) [79], which is comparable with this study (0.0063% *v*/*v*). In addition, it was shown that linalool inhibits the angiogenic activity of endothelial cells by downregulating intracellular ATP levels and activating TRPM8 [80].

Support of proliferation and fibroblast viability by thymol (besides activating the caspase system) is the potential activation of FYVE domain-containing protein 2, a known activator of Akt 2 kinase, which takes part in fibroblast migration wound healing and proliferation [81]. Like thymol, neral and geranial as major VOCs of MO showed several targets promoting cell viability processes in the STITCH database and demonstrated antitumor activity [82] and anti-inflammatory effects in murine macrophages [83]. In addition, *α*-terpineol also exerts anti-cancer activity, partly mediated by the suppression of NF-κB [84].

In contrast, the situation with camphor protein networking and potential effects on fibroblast proliferation is not straightforward (Figure 5). Group II metabotropic glutamate receptors (GRM2) signalling could cause a slowdown of the cell cycle by inhibiting adenylyl cyclase, reducing the cell cAMP and consequently reducing protein kinase C activity, which in turn will lead to a decreased level of T cell proliferation [85] or decreased vascular smooth muscle cell proliferation [73]. In addition, the regulator of G protein signalling 6 (RGS6) possesses potent pro-apoptotic and growth-suppressive actions [86]. Its activation would, therefore, presumably lead to a reduced proliferation and growth rate. Opposite to this, in many cell types, cAMP inhibits the physiological actions of growth factors mediated by G protein pathways that regulate cAMP and the cAMP-dependent protein kinase PKA [87].

Furthermore, activated PKA prevents the Raf kinase activation required for ERK1/2 activation and proliferative responses in fibroblasts [88]. The result of decreased cAMP by the activation of the receptor for peptide YY (NPY1R) (Figure 5) and G alpha (i) protein signalling that inhibits adenylate cyclase would, therefore, decrease the activity of PKA and lead to enhanced proliferation and fibroblast growth. In support of this, the results of Tran et al. [35] demonstrate the proliferative activities of pure camphor solution and vacuum-obtained camomile hydrolate with camphor as a major VOC in human primary dermal fibroblasts. Based on these findings, we can conclude that camphor is not the major VOC responsible for the slight cytotoxicity exerted by RO hydrolate.

Like camphor, menthol shows divergent protein targets in the STITCH primary protein network (Figure S3). Non-neuronal kappa-opioid receptor (OPRK1) activation enhances the proliferative capabilities of epidermal keratinocytes [89]. Activating transcription factor 3 (ATF3) can promote cancer cell proliferation or, on the other hand, act as a repressor of transcription [90]. As mentioned previously, menthol activates TRPM8, also known as cold and menthol receptor 1, and this leads to the inhibition of β1 integrin/FAK signalling, which in turn suppresses migration and might affect the proliferation as well, and it was found to promote wound healing in the Wistar rat model [91]. However, we cannot completely exclude menthol as not being responsible for the cytotoxic effects exerted by RO and LO hydrolates on dermal fibroblasts until explicit experiments with relevant menthol concentrations are conducted.

There are no data for borneol and its protein networking (accessed on the 10 April 2023, http://stitch.embl.de/cgi/network.pl?taskId=KgvdowNXhfdO). However, several publications reported this molecule’s cell growth having inhibitory and anti-proliferative actions [92,93], reducing proliferation and collagen matrix deposition in primary mice oral fibroblasts [94]. While the latter can be exploited as a desired effect for treating oral sub-mucous fibrosis in stomatology [94], it would certainly not be a wanted outcome in regular skin health and beauty maintenance. Terpinen-4-ol data (Figure S3) extracted from STITCH do not point to a protein target that could affect dermal fibroblast viability and proliferation. However, terpinene-4-ol was recognised as the *Melaleuca alternifolia* essential oil component that is responsible for antitumor and pro-apoptotic activity [95]. Thus, it could contribute to the observed cytotoxicity effects shown in Figure 4. The first-line protein networking of 1,8-cineole points to TRPM8 activation as a relevant protein target that could affect fibroblast proliferative and growth processes (Figure S3). In support of this, 1,8-cineole largely inhibited platelet activation stimulated by glycoprotein VI agonists (collagen and cross-linked collagen-related peptides) at a concentration of less than 6 μM [96], which is at least one order of magnitude higher than its concentrations in RO and LI hydrolates when applied at a 10% share in fibroblasts culture medium.

There are two study limitations, which could be resolved in future work related to identification of uncharacterized VOCs (the determination rate in LO hydrolate was below 90%). The answers should be well searched within the minor VOCs or aqueous soluble molecules peculiar for specific hydrolates that show either a beneficial effect on the fibroblast proliferation or, oppositely, a slight cytotoxic effect. The determination of hydrophilic compounds, such as derivatives of fatty acids, amino acids, and other metabolites of non-terpene origin; ketones; esters; and glycosides using high-resolution LC-MS-MS could help minimise or resolve the mystery of the molecule(s) responsible for the positive and harmful biological effects observed. For example, assessing the presence of polyphenolics such as kaempferol in concentrations (≥100 nM or ≥30 ppb) sufficient enough to prevent apoptotic cell death pathways by inhibiting abnormal JNK activation in dermal fibroblasts [97] or their abnormal proliferation via the inhibition of TGF-β1/Smads signalling [98] is warranted. Finally, it would be interesting to couple comprehensive hydrolate content characterisation with non-targeted, shotgun mass spectrometry-based proteomics of treated fibroblasts to understand which signalling pathways were up- and down-regulated.

## 4. Conclusions

Nine hydrolates of the Lamiaceae family that were addressed in this study contain one or up to four major VOCs (≥10% of the extracted organic part). Based on their biological activity on primary skin fibroblasts from a healthy donor, they were divided into three groups (promoting cell viability group, cytotoxic group, and no net effect group of hydrolates). A beneficial viability profile was demonstrated via a moderate but significant increase in the proliferation of primary human skin fibroblasts induced by clary sage, oregano, lemon balm, and partially by thyme hydrolates. Lavender exerted a noticeable cytotoxic effect, and lavandin and rosemary were less effective at suppressing proliferative activities. Mentha and winter savoury have fallen into the group with no net effect, though Mentha showed reduced viability in the MTT test at the 10% and 5% concentrations. The promoting viability effects could likely be attributed to thymol in oregano (OV) and thyme (TV) hydrolates. In lemon balm (MO) hydrolate, these are most likely neral and geranial compounds. The enigmatic compound is clary sage (SS) hydrolate, which dominates in terms of increased viability and fibroblast proliferation; however, its effect can only be supported by alpha terpineol and not by linalool activities. The answers should be explored among other classes of molecules that are present in traces, such as the characterisation of other reducing species and/or specific minor VOC profiles. Compounds with clear associations to cytotoxic effects or suppress fibroblast growth are borneol, 1,8-cineole, and terpinen-4-ol, and they are contained in lavender, lavandin, and rosemary hydrolates, exerting cytotoxic effects. More research on menthol bioactivities is warranted for it to be excluded as not responsible for LO and RO’s cytotoxic effects.

In general, all tested hydrolates, if applied at the concentration of 2.5% in the cosmetic formulation, would be safe for longer (chronic) skin exposure. In comparison, caution is mandatory at a 10% concentration with lavender hydrolate and, in the second instance, with lavandin and rosemary hydrolates. In addition, no proteins greater than 5 kDa and no peptides below 5 kDa have been detected at 0.01 ppm and 0.1 ppm, respectively, within the nine hydrolates. If the opposite was the case, some characteristics of hydrolates could be ascribed or attributed to protein/peptide species. The antioxidant capacity and scavenging radical activity of hydrolates did not correlate well with their viability profiles and reducing sugar levels. These properties correlate strongly with their total phenolic content. Thus, phenolics are regarded as the main source of antioxidative characteristics. Pertinent to skin cosmetic formulations aimed for frequent and longer use, it is advisable to monitor the level of glucose content as values well above the physiological norm would negatively affect fibroblast proliferation and the capability of building extracellular matrices and optimal collagen deposition. Further research is needed to understand the mechanisms of hydrolates’ biological activity entirely and use pure compounds to resolve the individual roles of the most responsible components for the observed effects that seem relevant to the cosmetic and wound healing aspects.

## Figures and Tables

**Table 1:** Volatile organic components (VOCs) were identified in the extracted organic part of hydrolates using GC/MS and are represented as percentages.

Volatile Organic Components	RI	MP	RO	LO	TV	SS	SM	LI	OV	MO
3-methyl-2-butenal	772							0.1		
hexanal	800	0.1			0.1					0.1
dihydro-methyl-3(2H)-furanone	805	0.1								
isovaleric acid	826	0.1			0.1					
furfural	830	0.1			0.4				0.1	
2-methyl-butanoic acid	835	0.1			0.2				0.1	
3-methyl-cyclopentanone	841	0.3								
trans-2-hexenal	846	0.7			0.1				0.3	0.1
cis-3-hexenol	848				0.2	0.7			0.2	1.6
cis-2-hexenol	858					0.6				
n-hexanol	860	0.1			0.1	0.3		1.0		0.4
2,5-diethyltetrahydro-furan	895	0.2								
3-methyl-cyclohexanone	947	0.9		0.1						
4-methyl pent-2-enolide	949							0.1		
benzaldehyde	958	0.2								0.1
2-ethenyltetrahydro-2,6,6-trimethyl-2H-pyran	968							0.1		
1-octen-3-ol	975	0.2	0.2	0.2	2.5	0.1	0.7	0.9	4.5	1.2
6-methyl-5-hepten-2-one	982									2.7
3-octanone	984		3.0	0.1				-	0.2	
dehydro-1,8-cineole	989			0.1						
3-octanol	991	0.7	0.9	0.1	0.2		0.1		0.3	0.1
dehydroxy-cis-linalool oxide	1006			0.1						
1,4-cineole	1014			-				0.1		
para-cymene	1022			0.1	0.4		0.3		0.3	0.1
1,8-cineole	1028	**12.8**	**12.6**	2.9	0.6			**12.7**	0.6	0.2
benzyl alcohol	1031								0.1	
lavender lactone	1037			0.2						
benzene acetaldehyde	1042	0.2		0.1					0.2	0.3
cis-sabinene hydrate	1064		0.1							
cis-linalool oxide (furanoid)	1069	0.4		1.2	0.2	0.5		1.4	0.3	0.2
fenchone	1084	0.4	0.8	2.4						
trans-linalool oxide (furanoid)	1086				0.2	0.5		1.3	0.3	0.1
p-cymenene	1087				0.1					
linalool	1098	0.2	2.1	5.7	2.8	**63.3**	0.2	**26.0**	2.7	2.1
α-pinene oxide	1102									0.6
hotrienol	1102			1.7						
filifolone	1103		0.9							
cis-rose oxide	1109									0.1
endo-fenchol	1112		0.1	0.1						
phenyl ethyl alcohol	1112	0.1							0.1	
cis-p-menth-2-en-1-ol	1119								0.1	
chrysanthenone	1123		0.5							
α-campholenal	1124			0.1						
nopinone	1136							0.2		
trans-sabinol	1136							0.1		
trans-pinocarveol	1137			0.2	0.1					
trans-para-menth-2-en-1 ol	1137	0.2							0.1	
camphor	1142		**29.2**	4.0	0.3			7.1	0.1	0.6
isopulegol	1142	0.6								
camphene hydrate	1146				0.1					
menthone	1152	**14.4**	6.4	6.9						0.1
iso-pulegol	1153									0.1
trans-pinocamphone	1160		0.2							
iso-menthone	1162	8.9		1.9						
pinocarvone	1162		0.2							
δ-terpineol	1164	0.7								
iso-menthone	1164		2.1							
borneol	1165		**10.1**	**21.8**	7.1		0.3	**24.4**	2.0	1.1
cis-linalool oxide	1168							0.6		
trans-linalool oxide	1173							0.4		
menthol	1173	**30.6**	**17.8**	**20.1**	0.1	0.1				0.2
terpinen-4-ol	1176	7.3	0.7	1.8	2.8	0.3	0.9	**12.2**	6.5	0.8
iso-menthol	1182	0.4	0.2							
p-cymene-8-ol	1183				0.3				0.5	
cryptone	1185			1.5						
α-terpineol	1190	1.3	3.1	1.6	0.7	**26.8**	0.2	6.0	1.7	0.4
myrtenol	1195		0.6	0.3						
verbenone	1201		1.7	0.4				0.2		
β-cyclocitral	1219									0.1
nerol	1225					1.9				1.8
m-cumenol	1226			0.7						
citronellol	1227									1.0
pulegone	1238	8.4	2.9	3.9						
neral	1241									**22.6**
carvacrol methyl ether	1242								0.2	
carvone	1243			0.7				0.1		
car-3-en-2-one	1251			0.1						
piperitone	1252	1.3								
cis-anethole	1252		0.2							
geraniol	1257					4.6				2.5
methyl citronellate	1259									0.1
geranial	1271									**20.5**
trans-anethole	1285		0.7	0.7						
para-cymene-7-ol	1290			1.7						
menthyl acetate	1293	0.2	0.1	0.3						
thymol	1296		0.1	0.2	**73.6**	0.1	0.5		**12.3**	0.6
carvacrol	1301		0.4	0.9	4.4		**96.4**		**65.5**	**27.7**
methyl geranate	1325									0.1
piperitenone	1340	0.1	0.3							
eugenol	1357								0.1	0.1
trans-β-damascenone	1383									0.1
caryophyllene oxide	1581		0.1							0.8
humulene epoxide II	1607		0.1							
epi-α-cadinol (=τ-cadinol)	1639			0.7						0.2
α-muurolol (=torreyol	1644									0.1
α-cadinol	1653									0.3
cis-14-nor-muurol-5-en-4-one	1687			0.3						
α-bisabolol oxide A	1744								0.1	
TOTAL	%	92.3	98.4	85.9	97.7	99.8	99.6	95.0	99.5	91.9

RI—Retention index; MP—*M. piperita*; RO—*R. officinalis*; LO—*L. officinalis*; TV—*T. vulgaris*; SS—*S. sclarea*; SM—*S. montana* ssp. *variegate*; LI—*L. intermedia*; OV—*O. vulgare*; MO—*M. officinalis*. Bolded numbers denote substantial compounds (≥10%) within the volatile organic part of respective hydrolate.

**Table 2:** Antioxidant activity of hydrolates according to DPPH and ABTS antioxidant tests and their total phenolic and reducing sugar contents.

(#) Hydrolate	pH	Total Reducing Sugars (GluE)	Antioxidant Tests				Total Phenolics (aq) (GAE)
			DPPH		ABTS		
		mg/L	TEAC μM ± SD	(%)	TEAC μM ± SD	(%)	mg/L
(1) **MP**	4.35 ± 0.05	445 ± 63	186 ± 2	(13 ± 0)	27 ± 10	(5 ± 2)	36.1 ± 1.3
(2) **RO**	6.30 ± 0.10	146 ± 12	40 ± 43	(4 ± 3)	57 ± 15	(10 ± 3)	35.9 ± 0.3
(3) **LO**	3.93 ± 0.02	305 ± 28	218 ± 65	(15 ± 4)	42 ± 10	(8 ± 2)	38.5 ± 0.7
(4) **TV**	4.33 ± 0.03	350 ± 17	755 ± 1	(49 ± 0)	465 ± 2	(89 ± 0)	204.2 ± 3.4
(5) **SS**	6.47 ± 0.04	664 ± 47	119 ± 70	(9 ± 4)	45 ± 5	(8 ± 1)	38.5 ± 2.0
(6) **SM**	7.03 ± 0.03	137 ± 18	566 ± 52	(37 ± 3)	471 ± 3	(90 ± 1)	194.2 ± 6.7
(7) **LI**	3.90 ± 0.01	1247 ± 99	200 ± 63	(14 ± 4)	20 ± 12	(3 ± 2)	38.5 ± 1.3
(8) **OV**	5.00 ± 0.02	204 ± 22	555 ± 64	(36 ± 4)	472 ± 3	(90 ± 1)	141.4 ± 2.0
(9) **MO**	6.70 ± 0.05	430 ± 40	222 ± 94	(15 ± 6)	197 ± 2	(37 ± 0)	50.9 ± 1.3

GluE—Glucose equivalents; aq—aqueous extraction; DPPH—2,2-diphenyl-1-picrylhydrazyl assay; ABTS—2,2′-azino-bis(3-ethylbenzothiazoline-6-sulfonic acid); TEAC—Trolox equivalent antioxidant capacity; GAE—gallic acid equivalents.

## Data Availability

The data presented in this study are available in the article itself or in the Supplementary Materials.

## Supplementary Materials

The following supporting information can be downloaded at: https://www.mdpi.com/article/10.3390/antiox12111988/s1, Table S1: Percentage of volatile organic compounds in hydrolates; Table S2: Correlation matrix for antioxidative properties by DPPH and ABTS+ tests and total phenolic content of nine hydrolates; Table S3: Protein and peptide concentration determination via a bicinchoninic acid assay; Figure S1: Polyacrylamide gel with samples obtained by a TCA/acetone protein precipitation method from nine hydrolates; Figure S2: Proliferation of fibroblasts treated with recombinant human epidermal growth factor; Figure S3: The confidence view of the protein networking of menthol, terpinene-4-ol, and 1,8-cineole (representatives of the major VOCs from hydrolates that exert negative viability effect), performed with the STITCH database http://stitch.embl.de/.
