# Supplementary material for: Multistep Approach Points to Compounds Responsible for the Biological Activity and Safety of Hydrolates from Nine Lamiaceae Medicinal Plants on Human Skin Fibroblasts
Source: Antioxidants (Basel). 2023 Nov 9;12(11):1988. doi: 10.3390/antiox12111988 (PMC10669667; doi:10.3390/antiox12111988)
Supplement: Supplementary file 1 [file antioxidants-12-01988-s001.zip › antioxidants-2682939-supplementary.pdf]

Supplementary Information

Multistep Approach Points to Compounds Responsible for the Biological Activity and Safety of Hydrolates from Nine *Lamiaceae* Medicinal Plants on Human Skin Fibroblasts

Katarina Smiljanić [1,*], Ivana Prodić [2], Sara Trifunovic [3,4], Maja Krstić Ristivojević [1], Milica Aćimović [5], Jovana Stanković Jeremić [6], Biljana Lončar [7] and Vele Tešević [1]

1 University of Belgrade—Faculty of Chemistry (UBFC), Studentski Trg 12–16, 11158 Belgrade, Serbia; krstic_maja@chem.bg.ac.rs (M.K.R.); vtesevic@chem.bg.ac.rs (V.T.)
2 Institute of Virology, Vaccines and Sera "Torlak"—National Institute of the Republic of Serbia, Vojvode Stepe 458, 11152 Belgrade, Serbia; iprodic@torlak.rs
3 Institute of Molecular Genetics and Genetic Engineering (IMGGE), University of Belgrade, Vojvode Stepe 444a, 11042 Belgrade, Serbia; strifunovic@imgge.bg.ac.rs
4 Mediterranean Institute for Life Sciences, 21000 Split, Croatia
5 Institute of Field and Vegetable Crops—National Institute of the Republic of Serbia, 21101 Novi Sad, Serbia; milica.acimovic@ifvcns.ns.ac.rs
6 Institute of Chemistry, Technology and Metallurgy, National Institute of the Republic of Serbia, University of Belgrade, 11000 Belgrade, Serbia; jovanas@chem.bg.ac.rs
7 Faculty of Technology Novi Sad, University of Novi Sad, 21000 Novi Sad, Serbia; cbiljana@uns.ac.rs
* Correspondence: katarinas@chem.bg.ac.rs

Katarina Smiljanić ORCID 0000-0003-4774-8895, katicas71@gmail.com
Sara Trifunović ORCID 0000-0002-3324-8016, strifunovic@imgge.bg.ac.rs
Maja Krstić ORCID 0000-0002-8794-9408, krstic_maja@chem.bg.ac.rs
Ivana Prodic ORCID 0000-0003-0604-9246, iprodic@torlak.rs
Milica Aćimović ORCID 0000-0002-5346-1412, milica.acimovic@ifvcns.ns.ac.rs
Jovana Stanković Jeremić ORCID 0000-0003-1560-5822, jovanas@chem.bg.ac.rs
Biljana Lončar ORCID 0000-0003-2994-6871, cbiljana@uns.ac.rs
Vele Tešević ORCID 000-0001-7060-9055, vtesevic@chem.bg.ac.rs

***Corresponding author:**
Katarina Smiljanić
Senior Research Associate
Centre of Excellence for Molecular Food Sciences and Department of Biochemistry, University of Belgrade — Faculty of Chemistry, Studentski Trg 12–16, 11158 Belgrade Serbia
Email: katarinas@chem.bg.ac.rs
Phone: +381 63 220360

Table S1: Percentage of volatile organic compounds in hydrolates

**Hydrolate**	**% of VOC content***
(1) MP - *M. piperita*	0.04 ± 0.01
(2) RO - *R. officinalis*	0.05 ± 0.02
(3) LO - *L. officinalis*	0.05 ± 0.02
(4) TV - *T. vulgaris*	0.11 ± 0.01
(5) SS - *S. sclarea*	0.04± 0.01
(6) SM - *S. montana* ssp. *variegata*	0.10 ± 0.02
(7) LI - *L. intermedia*	0.07 ± 0.02
(8) OV - *O. vulgare*	0.09 ± 0.03
(9) MO - *M. officinalis*	0.06 ± 0.01

*Values represent average value of triplicates ± SD.

Table S2. Correlation matrix for antioxidative properties by DPPH and ABTS+ test and total phenolic content of nine hydrolates

**Correlation Pearson r**	DPPH	ABTS+	TPC	**Correlation P values**	DPPH	ABTS+	TPC
DPPH		0.927	0.960	DPPH	0.0003	0.0003	0.0004
ABTS+	0.927		0.952	ABTS+			0.0007
TPC	0.960	0.952		TPC	0.00004	0.0001	

Legend: r – Pearson correlation coefficient; ABTS+ - antioxidative test; DPPH - 2,2-diphenyl-1-picrylhydrazyl antioxidative test; TPC – total phenolic content determination.

Table S3. Protein and peptide concentration determination via micro and macro method of bicinchoninic acid (BCA) assay

**Method**	**Standard curve with bovine serum albumin as standard (mg/mL)**	**Sample/Hydrolate**	**SpeedVac concentrated hydrolates samples (Fig. 1) (mg/mL)**	**TCA/acetone precipitated samples (Fig. S2) (mg/mL)**
	Blank (zero)	MP - *M. piperita*	as blank	as blank
micro	0.0025	RO - *R. officinalis*	as blank	as blank
	0.005	LO - *L. officinalis*	as blank	as blank
	0.01	TV - *T. vulgaris*	as blank	as blank
	0.02	SS - *S. sclarea*	as blank	as blank
macro	0.04	SM - *S. montana* ssp	as blank	as blank
	0.08	LI - *L. intermedia*	as blank	as blank
	1.20	OV - *O. vulgare*	as blank	as blank
	1.60	MO - *M. officinalis*	as blank	as blank
		Ambrosia extract 2x diluted	N/A	1.25

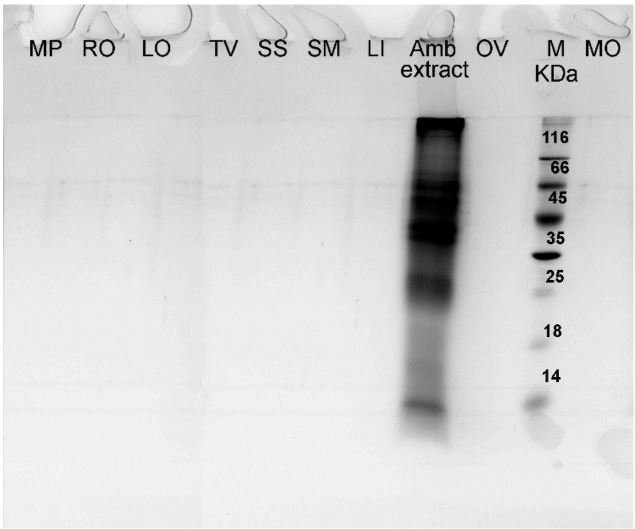

Figure S1. Polyacrylamide gel with samples obtained by TCA/acetone protein precipitation method from nine hydrolates. Electrophoretically resolved sodium dodecyl sulphate polyacrylamide gel (16%), with 10 mL of hydrolates precipitated with 40 mL 13% TCA/acetone overnight at -20 °C vacuum) per protocol described by Sheoran et al. [48]. Tentative pellet was mixed with 20 µL of 1 × Laemmli buffer in denaturing conditions, stained with CBB 250-R. Protein markers were the quantitative type of control, while protein extract obtained in house from the Ambrosia artemissfolia pollen, was used as a positive protein control (10 µL of 2,5 mg/mL). *M. piperita* (MP), *R. officinalis* (RO), *L. officinalis* (LO), *T. vulgaris* (TV), *S. sclarea* (SS), *S. montana* ssp. *variegata* (SM), *L. intermedia* (LI), *O. vulgare* (OV) and *M. officinalis* (MO), M – protein weight markers in kilo Daltons (kDa).

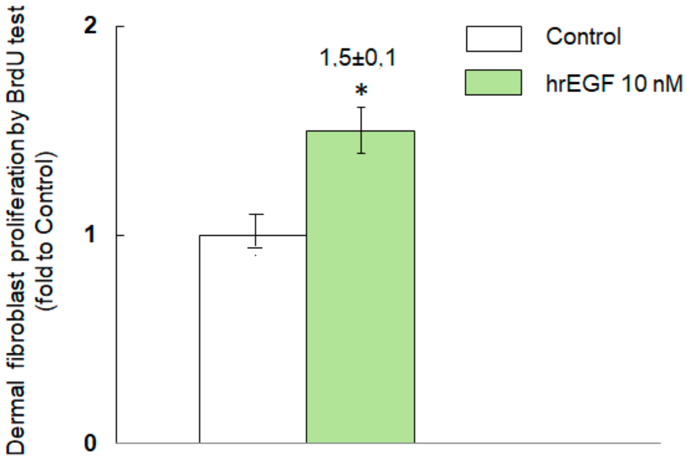

Figure S2. Proliferation of primary human dermal fibroblasts treated with 10 nM recombinant human epidermal growth factor (hrEGF) during 48 h, assessed through DNA synthesis via BrdU testing. *denotes significantly different proliferation at p< 0.05, compared to the control group.

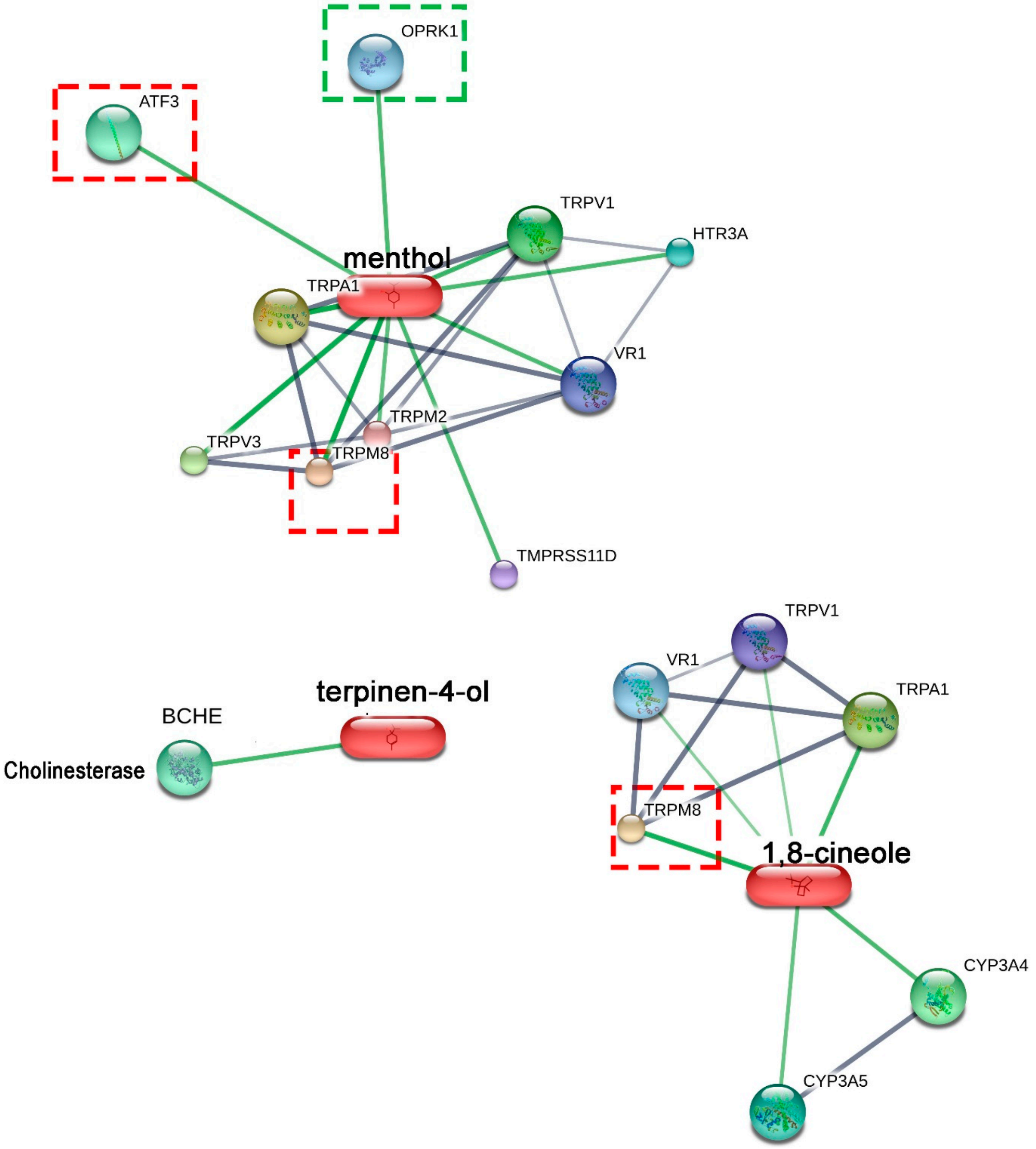

Figure S3. The confidence view of protein networking of menthol, terpinene-4-ol and 1,8-cineole (representatives of the major VOC from hydrolates that exert negative viability effect), done by STITCH database http://stitch.embl.de/. Only first-line protein targets of chemical compounds are shown with the host species of *Homo sapiens*.

References

Sheoran, I.; Ross, A.; Olson, D.; Sawhney, V. Compatibility of plant protein extraction methods with mass spectrometry for proteome analysis. *Plant Science* **2009**, *176*, 99-104, doi:10.1016/j.plantsci.2008.09.015.
